# Dislocation of the Shoulder of Long Standing—A Clinical Lecture

**Published:** 1888-02

**Authors:** J. McFadden Gaston

**Affiliations:** Professor of Surgery in the Southern Medical College, Atlanta, Ga.


### Southern Medical Record.

A MONTHLY JOURNAL OF PRACTICAL MEDICINE.

☞All Communications must be addressed to R. C. WORD, M. D., Managing Editor.

"*Quicquid Præcipies Esto Brevis.*"

VOL. XVIII. ATLANTA, GA., FEBRUARY, 1888. No. 2.

### ORIGINAL AND SELECTED ARTICLES.

### DISLOCATION OF THE SHOULDER OF LONG STANDING—A CLINICAL LECTURE.

BY J. MCFADDEN GASTON, M.D.,
Professor of Surgery in the Southern Medical College, Atlanta, Ga.

*Gentlemen:*

The case presented to-day is a dislocation of the humerus with the scapula, causing, as you perceive, a characteristic deformity of the shoulder. This man has come for examination, and if it is thought practicable to afford any relief to his sufferings, or to remedy the consequences of this displacement of the head of the bone, he is disposed to submit to any operation which may be requisite. Although I have not previously seen the patient, a statement has been made to me by a friend of his, that no effort was made immediately after the accident to reduce the dislocation, and that a dressing was applied by his medical attendant under the impression that there was a fracture of the neck of the bone.

If Dugas' proceeding had been instituted then, by carrying the hand of the injured arm to the opposite shoulder, it would have been found that the elbow could not at the same time have rested upon the side of the body, thus signifying the existence of the dislocation. The depression below the acromion process must, however, have been so marked from the outset that any surgeon who had ever read of such a departure from the normal relation of these parts should have been able to recognize the nature of the injury, even if he had never seen a case previously. You observe now that there exists quite a striking gap between the upper part of the humerus and the acromion process with a notable saliency of the point of the latter. This is rendered more distinct owing to the atrophy of the deltoid muscle from comparative inaction, and you perceive the great prominence of the head of the humerus beneath the corocoid process of the scapula indicating clearly an anterior downward dislocation. Upon seizing the elbow with one of my hands, while the other grasps the wrist with the forearm at right angles to the humerus of the patient, a sweep of the hand back and forth imparts rotation to the shaft of the bone which lifts and depresses the head of the humerus so that you can detect its movements readily from your seats. It is found upon searching for the site of the axillary artery that the rounded head has passed in advance of it, and doubtless also of the nerve which lies near it, so that these structures were lodged behind the neck of the humerus where it escaped from the articular capsule, and, hence, must have contracted intimate adhesions with the adjacent tissues. You will readily understand, therefore, that the amount of force requisite to restore the head of the humerus to its proper position must risk laceration of the artery; and further that the glenoid cavity has been so completely filled by admitting tissues after the lapse of two years, that it would be extremely doubtful whether the head of the bone could be kept in its position if it were practicable to dislodge it from its present site with safety. The only plan that seems at all feesible is that of making a dissection around the head of the humerus so as to detach it from the artery and nerve, and then opening up the glenoid cavity by cutting away the new formation which fills it, preparatory to carrying the head of the bone into its original location. But the transaction, involving such important structures, would most probably lead to serious results, and should not be risked while there is a prospect of some useful employment of the limb in its present condition.

When the patient is able to move his arm with comparative freedom, as you see him do, forward and backward, with a limited capacity upward; having, as I find by getting him to grasp my hand, a fair power of contraction in the flexor muscles, it is the part of prudence not to undertake any operative measure which may compromise this usefulness of the arm or endanger the life of the patient.

Notwithstanding that he suffers from numbness in the limb and neuralgia of the shoulder, for which he is now under treatment by massage and electricity, without much prospect of any permanent relief, I must enjoin upon him to suffer his present ills rather than rush upon others of which he cannot foresee the consequences.

Some of your number were present when I made an effort to reduce an anterior and downward dislocation of the shoulder of ten weeks' standing, about eighteen months ago, with the assistance of Dr. W. S. Elkin. We placed the man under anæsthesia, and resorted to longitudinal traction with the heel in the axilla; likewise to traction at a right angle with the body, while a towel around the thorax was held by assistants as a means of counter extension, making such manipulations as might aid in breaking up the adhesions. The arm was in each instance, after continued extension, carried across the chest with upward pressure of the elbow, with a view to restore the head of the humerus. But all proved unavaling after the use of such force as was thought safe, and the patient awoke from the anæsthetic to realize that the defect had not been relieved, being assured at the same time that there was no prospect of remedying the dislocation without great risk of lacerating the artery and injuring the nerve.

You will understand that this case, and others of a similar nature, should influence me in pursuing a course of inaction in the case brought before you to-day, after remaining displaced for two years, without any prospect of benefit.

I have been called in consultation within the past two weeks, by Dr. E. J Roach, in a case of sub-corocoid dislocation of the humerus on the left side, which had remained displaced for six weeks. By an energctic use of the same measures which failed in the case of ten weeks' standing, we succeeded in reducing this dislocation, and thus affording encouragement for attempting replacement under similar circumstances.

